# Bioenergy and Its Environmental Impacts

**DOI:** 10.1155/2015/508037

**Published:** 2015-05-07

**Authors:** Shungui Zhou, Yang-Chun Yong, Bin Cao, Hu-Chun Tao, Li Zhuang

**Affiliations:** ^1^Guangdong Institute of Eco-Environmental and Soil Sciences, Guangzhou 510650, China; ^2^School of The Environment, Jiangsu University, 301 Xuefu Road, Zhenjiang 212013, China; ^3^School of Civil & Environmental Engineering and Singapore Centre on Environmental Life Sciences Engineering, Nanyang Technological University, 50 Nanyang Avenue, Singapore 639798; ^4^School of Environment & Energy, Peking University, Nanshan District, Shenzhen 518055, China

Bioenergy is renewable energy and offers the promise for reducing dependency on fossil fuels. The main environmental impacts of bioenergy are associated with land use and greenhouse gas emission. This special issue covers multidisciplinary approaches and intensive research orientations for the real challenges in bioenergy and its environmental impacts. For this special issue, we received 35 submissions from all over the world. After an initial screening, 6 submissions were declared “out of scope” and the remaining 29 were sent to reviewers. All manuscripts underwent a very rigorous peer review process. We finally selected 12 full papers and 1 review paper for this special issue. In this issue, bioenergy from algae is highly concerned, including oil production from microalgae, life cycle fossil energy ratio of algal biodiesel, microbial fuel cell (MFC) with photosynthetic algae cathode, and algal biofuel production with wastewater. MFC, a new technology for electricity generation from waste and biomass, is popular in this special issue. The authors reported that the use of nanostructured manganese oxide as cathodic catalyst and the use of carbon nanofiber modified graphite felt as anode can significantly enhance MFC performance. The review paper focused on the state-of-the-art method for assessing the greenhouse gas (GHG) emission reduction by developing energy crops and a new approach combining life cycle analysis (LCA) with biogeochemical process models to assess GHG emission reduction. Although research on bioenergy has increased in recent years, the study of developing and screening the most suitable and economically viable technology needs much further attention.

